# Incidence of new onset cancer in patients with a myocardial infarction – a nationwide cohort study

**DOI:** 10.1186/s12872-018-0932-z

**Published:** 2018-10-22

**Authors:** Morten Malmborg, Christine Benn Christiansen, Michelle D. Schmiegelow, Christian Torp-Pedersen, Gunnar Gislason, Morten Schou

**Affiliations:** 1 Department of Cardiology, Herlev and Gentofte Hospital, Hjertemedicinsk forskning 1, Kildegårdsvej 28, opg. 8, 3.sal tv, DK-2900 Hellerup, Denmark; 2 Aalborg University Hospital, Aalborg, Denmark; 3 Institute of Health Science and Technology, Aalborg University, Aalborg, Denmark; 4 Danish Heart Foundation, Copenhagen, Denmark

**Keywords:** Myocardial infarction, Cancer, Epidemiology, Patient registries

## Abstract

**Background:**

Few studies have suggested that patients with myocardial infarction (MI) may be at increased risk of cancer, but further large register-based studies are needed to evaluate this subject. The aim of this study was to assess the incident rates of cancer and death by history of MI, and whether an MI is independently associated with cancer in a large cohort study.

**Method:**

All Danish residents aged 30–99 in 1996 without prior cancer or MI were included and were followed until 2012. Patients were grouped according to incident MI during follow-up. Incidence rates (IR) of cancer and death in individuals with and without MI and incidence rate ratios (IRR, using multivariable Poisson regression analyses) of cancer associated with an MI were calculated.

**Results:**

Of 2,871,168 individuals, 122,275 developed an MI during follow-up, 11,375 subsequently developed cancer (9.3%, IR 19.1/1000 person-years) and 65,225 died (53.3%, IR 106.0/1000 person-years). In the reference population, 372,397 developed cancer (13.0%, IR 9.3/1000 person-years) and 753,767 died (26.3%, IR 18.2/1000 person-years). Compared to the reference population, higher IRs of cancer and death were observed in all age groups (30–54, 55–69 and 70–99 years) and time since an MI (0–1, 1–5 and 5–17 years) in the MI population. MI was associated with an increased risk of overall cancer (IRR 1.14, 95% CI 1.10–1.19) after adjusting for age, sex and calendar year, also when additionally adjusting for chronic obstructive pulmonary disease, hypertension, dyslipidemia, diabetes and socioeconomic status (IRR 1.08, 95% CI 1.03–1.13), but not after further adjustment for the first 6 months post-MI (IRR 1.00, 95% CI 0.96–1.05).

**Conclusion:**

Patients after an MI have increased incidence of cancer, which may be explained by mutual risk, occult cancers and increased surveillance. Focus on risk factor management to reduce cancer and MI is warranted.

**Electronic supplementary material:**

The online version of this article (10.1186/s12872-018-0932-z) contains supplementary material, which is available to authorized users.

## Background

Ischemic heart disease and cancer are the two primary causes of death worldwide. Denmark is the only country in Europe where cancer has surpassed cardiovascular disease as the primary cause of death in both women and men [1, 2]. Due to improved management of MI patients such as increased use of prophylactic pharmacotherapy and more available use of cardiac revascularization, mortality rates after an MI, short-term as well as long-term, have decreased over the past decades [3, 4]. Consequently, the increasing number of MI survivors are at risk of dying from non-cardiac competing causes of death, cancer in particular, as has previously been demonstrated for STEMI patients following percutaneous coronary intervention [5, 6] and among patients with heart failure [7].

The association between cancer and MI is biologically plausible due to shared pathogenesis such as oxidative stress [8, 9] and shared risk factors (i.e. tobacco, alcohol consumption, obesity, physical inactivity, unhealthy food consumption and metabolic disorders such as hypertension, dyslipidemia and diabetes) [10, 11]. Additionally, occult cancers are prothrombotic [12] and increase the risk of MI [13]. However, the risk of cancer following an MI remains scarcely investigated. Although, three studies have found an increased risk of cancer after an MI, [14–16] of which the two oldest studies found an increased risk of predominantly tobacco-related cancers, none of them have taken the high mortality after an MI into account.

To evaluate the association between an MI and cancer there are a number of caveats that need to be accounted for, which involves analyses of competing risk and surveillance bias. Due to high mortality rates after an MI, an independent association between an MI and cancer may, therefore, have been overlooked in previous studies, and the possible association calls for further investigations.

Therefore, the aims of this study were to assess the incident rates of cancer and death by history of MI, as well as the association between an MI and new onset cancer in patients surviving a first-time MI compared with the reference population.

## Methods

### Data sources

All residents in Denmark receive a unique and permanent civil registration number at birth or immigration that enables linkage between nationwide registers at an individual level. Data for this study were obtained from four large Danish registries: The Danish Civil Registration System registry, the Danish National Patient Registry (NPR), the National Causes of Death Registry and the Danish National Prescription Registry. From the Danish Civil Registration System registry we obtained information on sex, date of birth and vital status (recorded within two weeks from the event), whereas causes of death were obtained from the National Causes of Death Registry and classified according to the International Classification of Diseases (ICD)-8 until 1993 and ICD-10 from 1994 and onwards. From the National Patient Registry we achieved information on all hospital admissions since 1977 with diagnoses coded according to the ICD system. Lastly, we obtained information on all claimed prescriptions in Denmark since 1995 from the Danish National Prescription Registry according to the Anatomical Therapeutic Chemical (ATC).

### Study population

Our study population comprised all individuals aged 30–99 years residing in Denmark on 1 January 1996. Patients with a first-time diagnosis of MI (ICD-8 code 410, ICD-10 codes I21–22) or first-time cancer (ICD-8 codes 140–209, ICD-10 codes C00–96) prior to 1 January 1996 were excluded, as were individuals who had a first-time diagnosis of cancer and MI on the same day during follow-up. Patients were grouped according to incident MI during follow-up. Thus, the reference population comprised the total Danish population without prior MI or cancer in 01 January 1996, and the MI population comprised those individuals from the reference population who developed an MI during follow-up without prior cancer at the time of an MI. The ICD-8 diagnosis of an MI in the NPR has been validated in the Danish MONICA (Monitoring Trends and Determinants in Cardiovascular Disease) study with a positive predictive value of 93.5% for definite or possible MI and a sensitivity of 92.8% for definite MI [17]. The ICD-10 diagnosis of MI as well as cancer in the NPR has been validated with a positive predictive value of 98% and 98–100%, respectively [18, 19].

The following comorbidities were identified: hypertension, dyslipidemia, diabetes and chronic obstructive pulmonary disease (ICD-8 codes 490–492 and ICD-10 codes J42, J44). Hypertension was defined as combination treatment with at least two classes of antihypertensive drugs within 90 days, as previously validated [20]. Diabetes mellitus was defined as the use of glucose-lowering medication (ATC A10), [21] and dyslipidemia as claimed prescriptions of lipid modifying agents (C10A). Medications at baseline were defined as dispensed prescriptions prior to inclusion for aspirin (B01AC06 and N02BA01), renin-angiotensin system inhibitors (C09AA, C09BA, C09BB, C09CA, C09DA, C09DB, C09XA02 and C09XA52), calcium channel blockers (C08, C07F, C09BB and C09DB), beta-blockers (ATC C07A-C07D and C07F) and statins (C10AA).

Socioeconomic status was identified as educational attainment and categorized as follows: (1) Basic education (primary, lower secondary); (2) upper secondary (general secondary, technical secondary); (3) vocational; (4) short or medium length higher education (Academy Profession Degree, Professional Bachelor’s Degree, university Bachelor’s Degree); (5) Master’s Degree or Ph.D. Degree; (6) unknown.

### Outcomes

The primary outcome was any incident cancer reported in the NPR or as cause of death (ICD-10 codes C00–96). From the NPR we also obtained information on the five most common types of cancer types according to the Association of Nordic Cancer Registries: Lung cancer including trachea and bronchus (C33–34), colorectal (C18–20), breast (C50), prostate (C61) and lower urinary tract cancer (LUT, ureters, bladder and urethra) (C66–68.0).

### Statistics

The reference population was followed from 1 January 1996 until an MI event, death, cancer event, emigration, age 100 or 31 December 2012, whichever came first, and the MI population was followed from diagnosis of MI until death, cancer event, emigration, age 100 or 31 December 2012, whichever came first.

We calculated crude incidence rates (IR) of cancer and death stratified by age groups (30–54, 55–69 and 70–99 years of age) and time since a diagnosis of MI (0–1, 1–5 and 5–17 years post-MI). We further examined the cumulative incidence of cancer according to history of an MI and age groups in which the competing risk of death was taken into account. We used a 0–1-2 outcome variable to determine those with no cancer diagnosis or date of death, those who received a cancer diagnosis or died from cancer, and those who died from other causes than cancer, respectively.

We used time-dependent multivariable Poisson regression models to examine the incidence rate ratios (IRR) of cancer associated with an MI, as previously performed [22]. Each individual contributed with disease-free exposure time until date of diagnosis of MI, and from this day onwards with time exposed for the disease. We used the lexis-macro [23] prior to all analyses to create three time scales: age (bands were split into 2-year intervals), calendar year (1-year intervals since January 1, 1996) and time following MI (1, 3, 6 and 12 months and every year hereafter). Dichotomous variables were created for MI (MI/no MI), and age was calculated at the beginning of each interval. Prior to the date of their first MI, individuals contributed risk time to the reference population, which in all analyses consisted of the general Danish population without MI.

We assessed the associations between MI and subsequent risk of cancer by two approaches. In the main analyses, we examined the association between MI and subsequent cancer risk according to time following MI (adjusted for sex, age and calendar year). Secondly, we explored the IRRs of cancer associated with MI stratified by age compared with risk of cancer in the reference population (adjusted for sex and calendar year). The same approach was used for the five selected types of cancers. All statistical analyses were conducted using SAS, version 9.4 (SAS institute, Cary, NC, USA).

### Sensitivity analyses

In accordance with our hypothesis, increased risk of cancer in MI patients could be confounded by shared risk factors or comorbidities that are markers of risk factors [24]. We, therefore, conducted sensitivity analyses, in which we adjusted for dyslipidemia, hypertension, diabetes mellitus, chronic obstructive pulmonary disease and socioeconomic status.

The same analyses were performed without the first six months following an MI, as this period of time is most likely to be affected by surveillance bias.

## Results

### Patient selection and study population characteristics

**Fig. 1:**
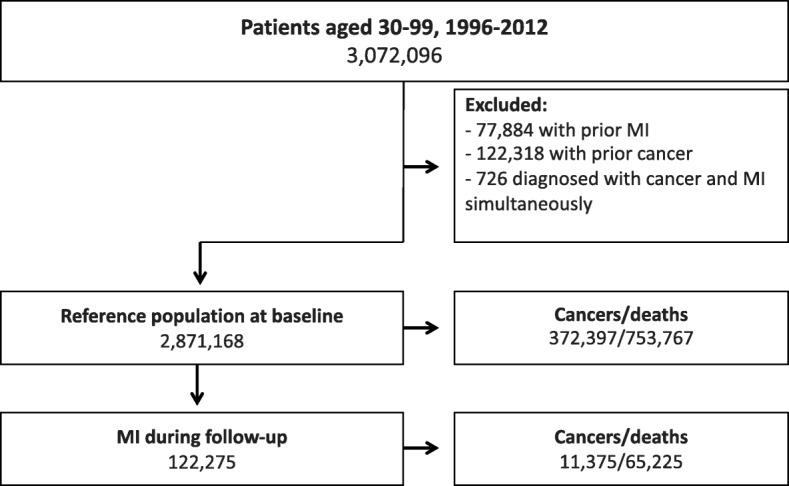
Flow chart of the study population. *MI* Myocardial infarction

Following exclusion of individuals according to the pre-defined criteria, the final population comprised 2,871,168 individuals without prior MI or cancer, and 122,275 individuals subsequently developed an MI (Fig. 1). At baseline, both men and women had a median age of about 50 years, whereas women were about 9 years older than men at time of first MI (Table 1). Individuals with MI were more often men and had a higher proportion of chronic obstructive pulmonary disease, hypertension, dyslipidemia and diabetes than the reference population at baseline.

**Table 1:** Baseline characteristics

	Reference population in 01 January 1996	MI population at time of MI
n	2,871,168	122,275
No. of person-years	40,038,194	594,061
Female, *n* (%)	1,493,795 (52.0)	47,501 (38.8)
Median age (IQR)		
Men	49.5 (39.5–61.3)	59.2 (49.5–69.5)
Women	51.7 (41.0–66.4)	68.5 (58.1–76.0)
Comorbidity, *n* (%)		
COPD	37,001 (1.3)	11,032 (9.0)
Dyslipidemia	13,794 (0.5)	21,408 (17.6)
Diabetes mellitus	58,495 (2.0)	15,154 (12.4)
Hypertension	116,249 (4.1)	17,187 (14.1)
Medication, n (%)		
Aspirin	121,922 (4.3)	44,145 (36.1)
Renin-angiotensin inhibitors	92,811 (3.2)	37,562 (30.7)
Calcium channel blockers	124,708 (4.3)	36,787 (30,1)
Beta blockers	121,792 (4.2)	33,771 (27.6)
Statins	10,638 (0.4)	21,200 (17.3)

*MI = myocardial infarction, IQR = interquartile range. COPD = chronic obstructive pulmonary disease*

### Risk of cancer and death, cumulative incidence functions and causes of death

**Fig. 2:**
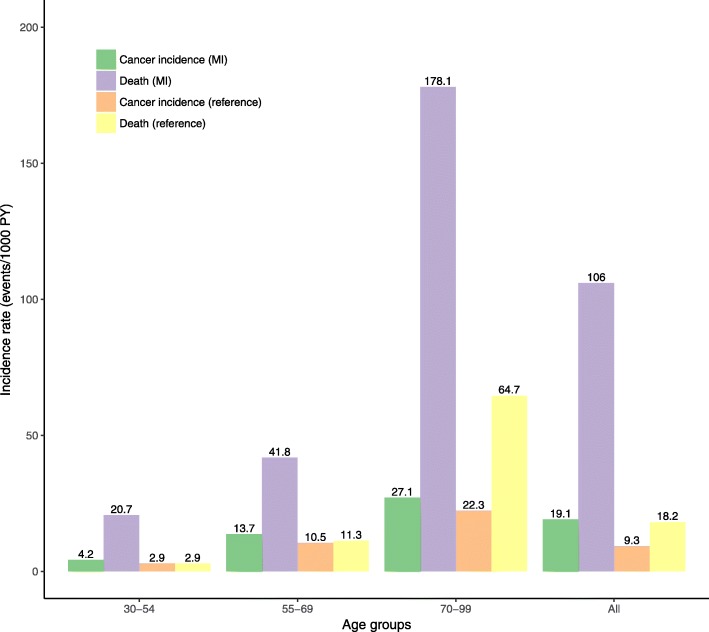
Incidence of cancer and death stratified by age group in the MI and reference population. 95% Confidence intervals from left to right: (3.8–4.7), (19.8–21.7), (2.8–2.9), (2.9–3.0), (13.2–14.2), (41.0–42.7), (10.5–10.6), (11.3–11.4), (26.5–27.7), (176.6–179.6), (22.2–22.4), (64.6–64.9), (18.8–19.5), (105.2–106.9), (9.3–9.3), (18.2–18.3). MI = Myocardial infarction

During mean follow-up, 11,375 (9.3%) of the MI patients developed first-time cancer and 65,225 (53.3%) died, whereas 372,397 (13.0%) individuals in the reference population developed cancer and 753,767 (26.3%) died (Fig. 1). The IRs of cancer and death increased with advancing age in both populations, but both cancer and death were more frequent among MI patients in all age groups, as well as time following MI (Fig. 2 and Additional file 1: Figure S1A-C). The highest incidences of death and cancer were observed during the first year following MI (Additional file 1: Figure S1A-C), but the incidence of death was higher than cancer, especially during the first year after an MI. In the reference population, the incidence of cancer was comparable to the incidences of death, except in the oldest individuals, in whom risk of death was higher than cancer risk (Fig. 2 and Additional file 1: Figure S1A-C). In MI patients, lung and prostate cancer were the most common subtypes of cancer, whereas breast cancer was the most common cancer in the reference population (Additional file 2: Table S1).

According to age and continuous time since MI, the cumulative incidence of cancer increased steadily, although the incidence rate for the oldest age group decreased over time and crossed the incidence rate of 55–69 year olds after approximately 7 years of follow-up (Additional file 1: Figure S2). In all age groups, cumulative incidence of death showed high mortality right after diagnosis of MI (Additional file 1: Figure S3).

### Relative risk of cancer

The highest risk of overall cancer was observed during the first month after an MI diagnosis with a subsequent decrease, reaching a somewhat constant level 6 months following a first time MI diagnosis (Additional file 2: Table S2A-B).

**Fig. 3:**
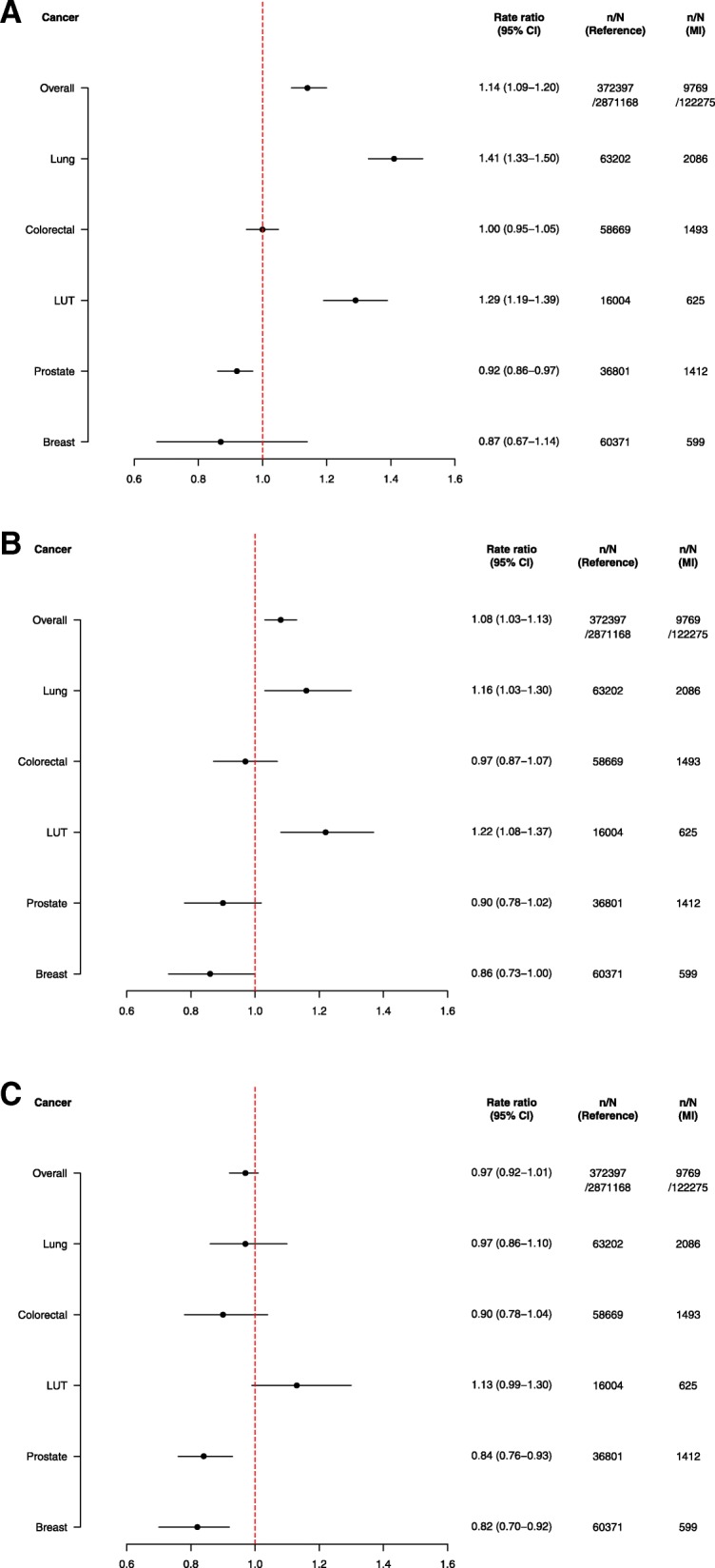
**a** Incidence rate ratios of overall cancer and selected subtypes of cancer associated with a myocardial infarction. Analyses are adjusted for age, sex and calendar year: MI = myocardial infarction, LUT = lower urinary tract, n = number of cancers, N = number of persons. **b**. Incidence rate ratios of overall cancer and selected subtypes of cancer associated with a myocardial infarction. Analyses are adjusted for age, sex, calendar year, hypertension, dyslipidemia, diabetes, chronic obstructive pulmonary disease and socioeconomic status. MI = myocardial infarction, LUT = lower urinary tract, n = number of cancers, N = number of persons **c**. Incidence rate ratios of overall cancer and selected subtypes of cancer associated with a myocardial infarction 6 months – 17 years post-MI. Analyses are adjusted for age, sex, calendar year, hypertension, dyslipidemia, diabetes, chronic obstructive pulmonary disease and socioeconomic status. MI = myocardial infarction, LUT = lower urinary tract, n = number of cancers, N = number of persons

MI patients had significantly increased risks of overall, lung and lower urinary tract cancer after adjustment for age, sex and calendar year (Fig. 3a), also after additional adjustment for diabetes, hypertension, dyslipidemia, chronic obstructive pulmonary disease and socioeconomic status (Fig. 3b). When further excluding the first 6 months following an MI, only lower urinary tract cancer remained significantly increased among MI patients (Fig. 3c). The risks of overall, lung, colorectal and lower urinary tract cancer were highest in the youngest age group (30–54) of MI patients and decreased with advancing age (55–69 years of age and 70–99 years of age, Additional file 2: Table S3A-C). Women were generally at increased risk of cancer compared to men (p for interaction < 0,0001), and the difference in risk of cancer attenuated after adjustment for comorbidities (Additional file 2: Table S4).

## Discussion

### Major findings

In this nationwide study, we observed that an MI is only modest associated with an increased risk of cancer after adjustment for traditional confounders, mutual risk factors and comorbidities and it became insignificant after exclusion of the first 6 months after the MI, suggesting that mutual risk factors, occult cancers and surveillance bias explain the increased incidence of cancer in the MI population.

### Incidence rates of cancer and death

The observation of increased cancer risk in the years following a diagnosis of MI is in accordance with previous studies [5, 6, 14–16] and age-stratified analyses did not change this observation. This finding may in part reflect surveillance bias and occult cancers in the early phase (0–1 year) after an MI, however, the surviving MI patients continued their lives at increased cancer risk compared with the reference population. It should be noted that since patients with a previous cancer diagnosis were excluded, end-stage cancer patients did not explain these findings. The cumulative incidence of cancer after an MI continued to increase over time in the two youngest age groups confirming that younger long-term survivors from an MI have an increased risk of cancer despite an increased risk of death compared to the reference population. The risk of cancer after an MI is, therefore, highest the first year after an MI, but despite a high risk of death, cancer continues to develop over time. A high mortality risk in the early years after an MI is, therefore, a larger clinical challenge than development of comorbidities such as cancer, and more research in mode of death and development of cardiovascular drugs for secondary prevention of cardiovascular death is still warranted.

### Relative risks of cancer (Poisson regression analyses)

The associations between a history of MI and cancer risk were largely attenuated following adjustment for comorbidities and became insignificant after adjustment for the first 6 months post-MI, suggesting that mutual risk factors, occult cancers and surveillance bias, and not a shared pathogenesis (e.g. oxidative stress between atherosclerosis and cancer), may explain the increased incidence of cancer after an MI. This is supported by the fact that the two smoke related cancers, lung and lower urinary tract cancer, seemed to be associated with MI before adjustment for comorbidities. Nonetheless, risk of lower urinary tract cancers remained significantly increased after full adjustment, which can likely be explained by unmeasured and residual confounding, e.g. history of tobacco. Based on our data, it is, however, difficult to explain the exact mechanism(s) why the association became insignificant after adjustment for comorbidities, but it may be speculated that environmental factors like tobacco and alcohol [25], unhealthy diet, shared genetic susceptibility, inflammation [26, 27] and medication play a role, since these mechanisms are reflected in the confounders adjusted for in the analyses. Furthermore, a previous study observed an increased incidence of cancer after MI in patients with heart failure compared to those without heart failure after MI [28]. Thus, eliminating comorbidities may have eliminated the sicker patients more prone to post-MI heart failure. Lastly, it should be mentioned that analyses in cumulative incidence showed that cancer may be even less of a concern in absolute measures than indicated by relative measures.

The strong associations between an MI and a new onset cancer in the first 6 months, especially the first month, even after adjustment for comorbidities compared to the remaining follow-up, indicate that our observations are likely explained by surveillance bias. Being in contact with the healthcare system increases the chance of early detection of cancer due to intensive clinical evaluation, a large number of tests and increased reporting of non-cardiac symptoms. Platelet inhibition may also induce bleeding in clinically silent cancers post MI, requiring further diagnostic testing. Another explanation to this association could be occult cancers during time of MI, as they provide a prothrombotic state [12] that may increase the risk of MI [13] and other arterial thrombotic diseases [29].

### Radiation and new onset cancer after an MI

One could speculate that MI patients are more exposed to radiation through percutaneous coronary interventions and other procedures, which could affect our results. If so, we would have expected increasing risk of developing cancers in the chest, such as lung and breast cancer, in the years following MI. In this study, breast cancer showed a non-significantly increased risk of cancer only 10–17 years following MI. However, after the first year following an MI, a history of an MI was consistently associated with increased risk of lung and lower urinary tract cancers, suggesting that smoking may be the main explanation to this observation.

### Strengths and limitations

The major strengths of this nationwide study include the diminished risk of selection bias, the minimal loss of follow-up ensured by the comprehensive Danish registries, and the large sample size of almost 3 million people with up to 17 years of follow-up, which gave us the unique possibility to study the risk of overall and subtypes of cancer in MI patients. Conversely, given the observational nature of this study, several important limitations need to be addressed.

The primary limitation was the risk of unmeasured and residual confounding by variables not available to us from the administrative registries, namely smoking, alcohol consumption, body mass index, physical activity level, diet and metabolic abnormalities not requiring medication. Further studies are needed to discover the effect of these common risk factors on the risk of cancer in MI patients. Secondly, surveillance bias is likely to have affected our results, thus being in contact with the healthcare system increases the chance of diagnosing a cancer. Thirdly, our results rely on discharge diagnoses on cancer and MI from hospitals. Nonetheless, although minor misclassifications exist in the NPR, especially when the coding practice is unclear, the misclassifications are non-systematic and do not influence the overall validity of the NPR data [19, 30–32]. Both the MI and cancer diagnoses have high predictive values and a high sensitivity. Theoretically some cases of an MI or a cancer may have been overlooked which may have biased our results toward zero. However, we do not think that misclassification of MI or cancer explain our results since our results are in accordance with previous studies on the association between atherosclerosis and incident cancer [14]. Whether the observed cancers within the first year after the MI are early stage cancers without impact on e.g. length of life cannot be addressed based on the present analyses. However, reverse causality may be a limitation given the higher risk of cancer in the first year and co-occurrence as seen in Fig. 1. Additionally, cancer events were primarily identified from NPR with supplement from the Danish Register of Causes of Death. We did not have access to The Danish Cancer Registry or the Danish Pathology Registry, and thus our study may underestimate the number of cancer events. However, the number is considered low, since the Danish Pathology Registry forwards information to the Danish Register of Causes of Death. Further studies are needed to examine whether more advanced cancers are more or less associated with MI. Furthermore, the definitions of some of the comorbidities in our data rely on information poorly registered in the registries. To accommodate this, in accordance with previous work, prescribed medications were used as proxies to define diabetes mellitus, hypertension and dyslipidemia [20, 21]. Lastly, the majority of the Danish population is white, so our results are not generalizable to non-white people.

## Conclusions

This study suggests that the increased incidence of cancer among patients with myocardial infarction may be due mutual risk factors, occult cancers and increased surveillance rather than an independent association. Furthermore, death is still a larger clinical challenge at all ages, especially by cardiac death rather than cancer in the early years after the myocardial infarction. Cardiologists and general practitioners should be aware of signs and symptoms of cancer in myocardial infarction survivors, but focus on secondary prevention and management after MI to reduce mortality is warranted.

## Additional files

Additional file 1: **Figure S1A.** Incidence rates of cancer and death stratified by age group 0-1 years post-MI in the reference and MI population. **Figure S1B.** Incidence of cancer and death stratified by age group 1-5 years post-MI in the reference and MI population. **Figure S1C.** Incidence of cancer and death stratified by age group 5-17 years post-MI in the reference and MI population. **Figure S2.** Cumulative incidence of cancer stratified by age group in the MI population. **Figure S3.** Cumulative incidence of death stratified by age group in the MI population. (DOCX 407 kb)

Additional file 2: **Table S1.** One-year incidence rates of subtypes of cancer per 1,000 person-years according to age group. **Table S2A.** Incidence rate ratios of cancer associated with an MI adjusted for age, sex and calendar year according to time since MI. **Table S2B.** Incidence rate ratios of cancer associated with an MI adjusted for age, sex, calendar year, dyslipidemia, hypertension, diabetes, chronic obstructive pulmonary disease and socioeconomic status according to time since MI. **Table S3A.** Incidence rate ratios of overall cancer and selected subtypes of cancer stratified by age group and adjusted for sex and calendar year. **Table S3B.** Incidence rate ratios of overall cancer and selected subtypes of cancer stratified by age group and adjusted for sex, calendar year, dyslipidemia, hypertension, diabetes, chronic obstructive pulmonary disease and socioeconomic status. **Table S3C.** Incidence rate ratios of overall cancer and selected subtypes of cancer 6 months – 17 years post-MI stratified by age group and adjusted for sex, calendar year, dyslipidemia, hypertension, diabetes, chronic obstructive pulmonary disease and socioeconomic status. **Table S4.** Incidence rate ratios of overall cancer and selected subtypes of cancer stratified by gender. (DOCX 54 kb)

## Abbreviations

ATC: Anatomical Therapeutic Chemical
ICD: International Classification of Diseases
IR: Incidence rates
IRR: Incidence rate ratio
LUT: Lower urinary tract
MI: Myocardial infarction
NPR: (Danish) National Patient Registry
