# Supplementary material for: Incidence of new onset cancer in patients with a myocardial infarction – a nationwide cohort study
Source: BMC Cardiovasc Disord. 2018 Oct 22;18:198. doi: 10.1186/s12872-018-0932-z (PMC6196475; doi:10.1186/s12872-018-0932-z)
Supplement: Supplementary file 1 — Figure S1A. Incidence rates of cancer and death stratified by age group 0-1 years post-MI in the reference and MI population. Figure S1B. Incidence of cancer and death stratified by age group 1-5 years post-MI in the reference and MI population. Figure S1C. Incidence of cancer and death stratified by age group 5-17 years post-MI in the reference and MI population. Figure S2. Cumulative incidence of cancer stratified by age group in the MI population. Figure S3. Cumulative incidence of death stratified by age group in the MI population. (DOCX 407 kb) [file 12872_2018_932_MOESM1_ESM.docx]

**Supplemental figures**

*Additional file 1: figure S1A. Incidence rates of cancer and death stratified by age group 0-1 years post-MI in the reference and MI population.*

*
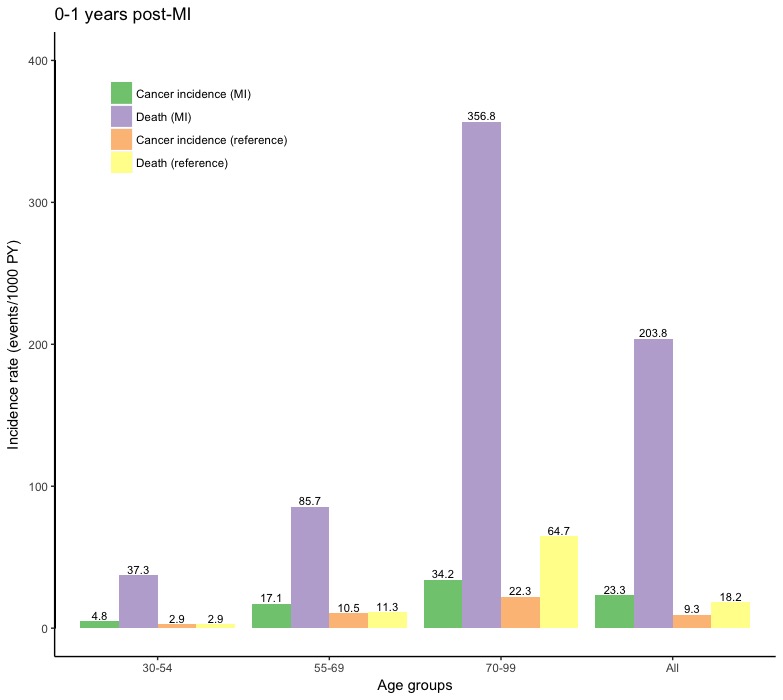
*

*95% Confidence intervals from left to right: (4.0-5.9), (35.2-39.6), (2.8-2.9), (2.9-3.0), (16.0-18.3), (83.4-88.0), (10.5-10.6), (11.3-11.4), (32.8-35.7), (352.8-360.9), (22.2-22.4), (64.6-64.9), (22.5-24.2), (201.7-206.0), (9.3-9.3), (18.2-18.3).
MI = Myocardial infarction.*

*Additional file 1: figure S1B. Incidence of cancer and death stratified by age group 1-5 years post-MI in the reference and MI population.*

*
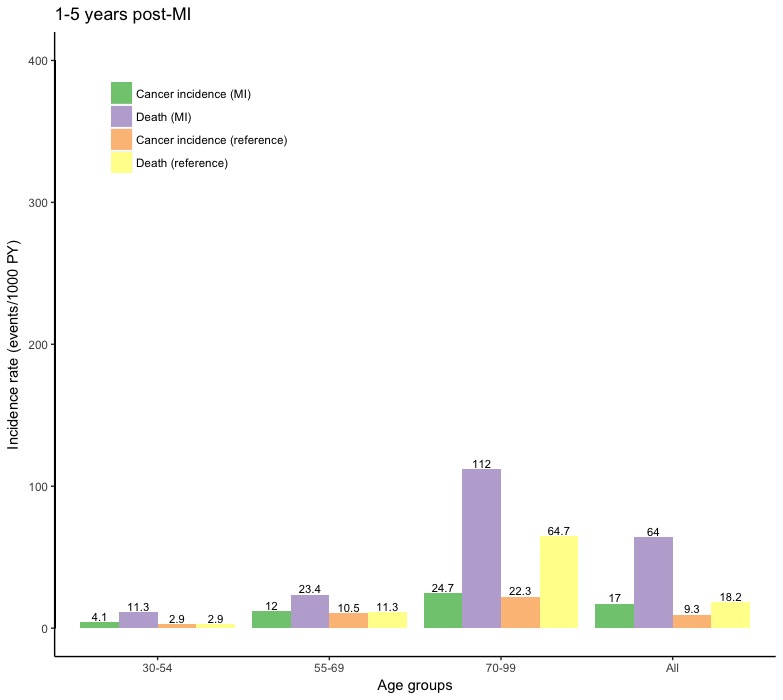
*

*95% Confidence intervals from left to right: (3.4-4.8), (10.3-12.4), (2.8-2.9), (2.9-3.0), (11.3-13.7), (22.4-24.4), (10.5-10.6), (11.3-11.4), (23.8-25.6), (110.1-114.0), (22.2-22.4), (64.6-64.9), (16.5-17.5), (63.0-65.0), (9.3-9.3), (18.2-18.3).
MI = Myocardial infarction.*

*Additional file 1: figure S1C. Incidence of cancer and death stratified by age group 5-17 years post-MI in the reference and MI population.*

*
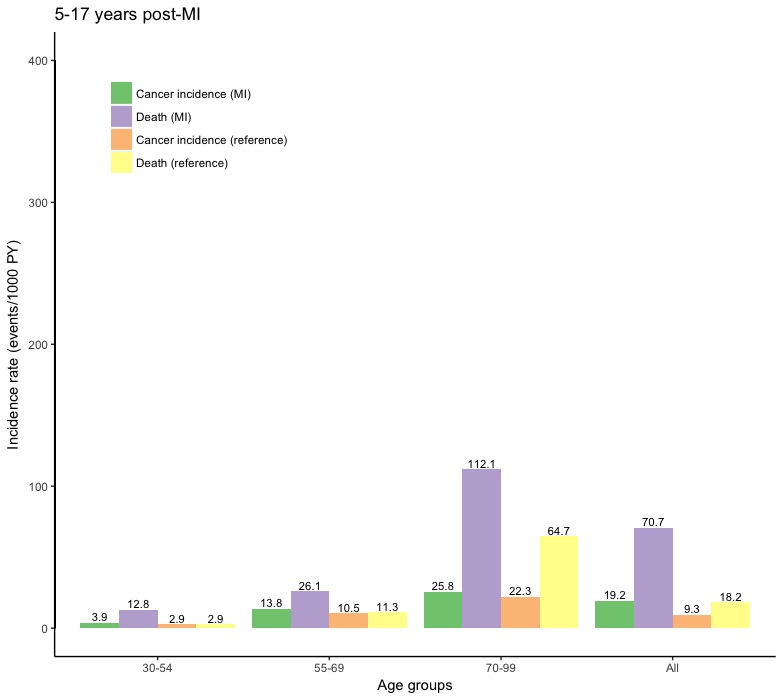
*

*95% Confidence intervals from left to right: (3.1-4.9), (11.3-14.6), (2.8-2.9), (2.9-3.0), (13.0-14.6), (25.0-27.3), (10.5-10.6), (11.3-11.4), (24.9-26.8), (110.1-114.1), (22.2-22.4), (64.6-64.9), (18.7-19.8), (69.6-71.9), (9.3-9.3), (18.2-18.3).
MI = Myocardial infarction.*

*Additional file 1: figure S2. Cumulative incidence of cancer stratified by age group in the MI population.*

*
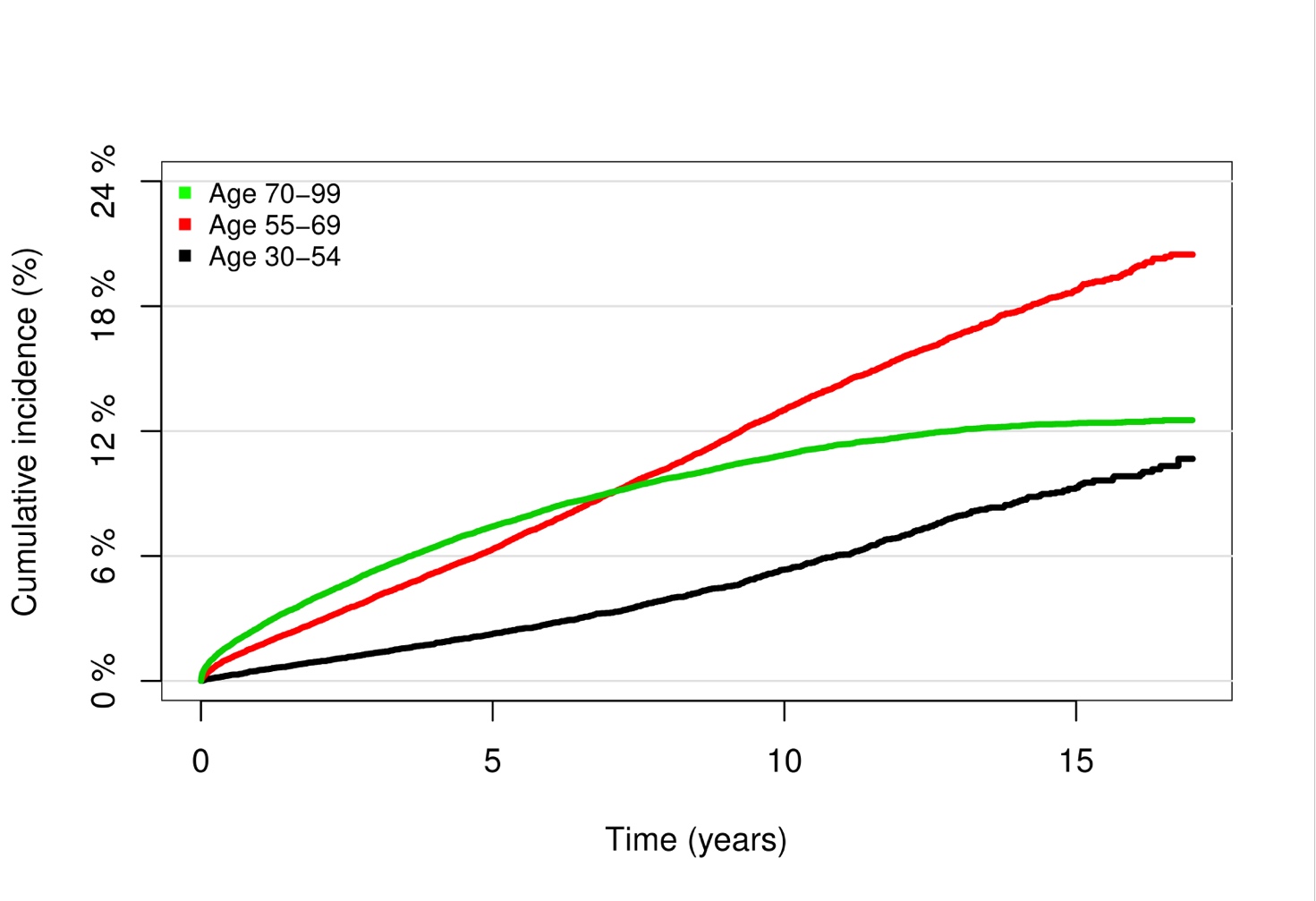
*

*N.B. The y-axis is narrower than in Supplemental figure 3.*

*Additional file 1: figure S3. Cumulative incidence of death stratified by age group in the MI population.*

*
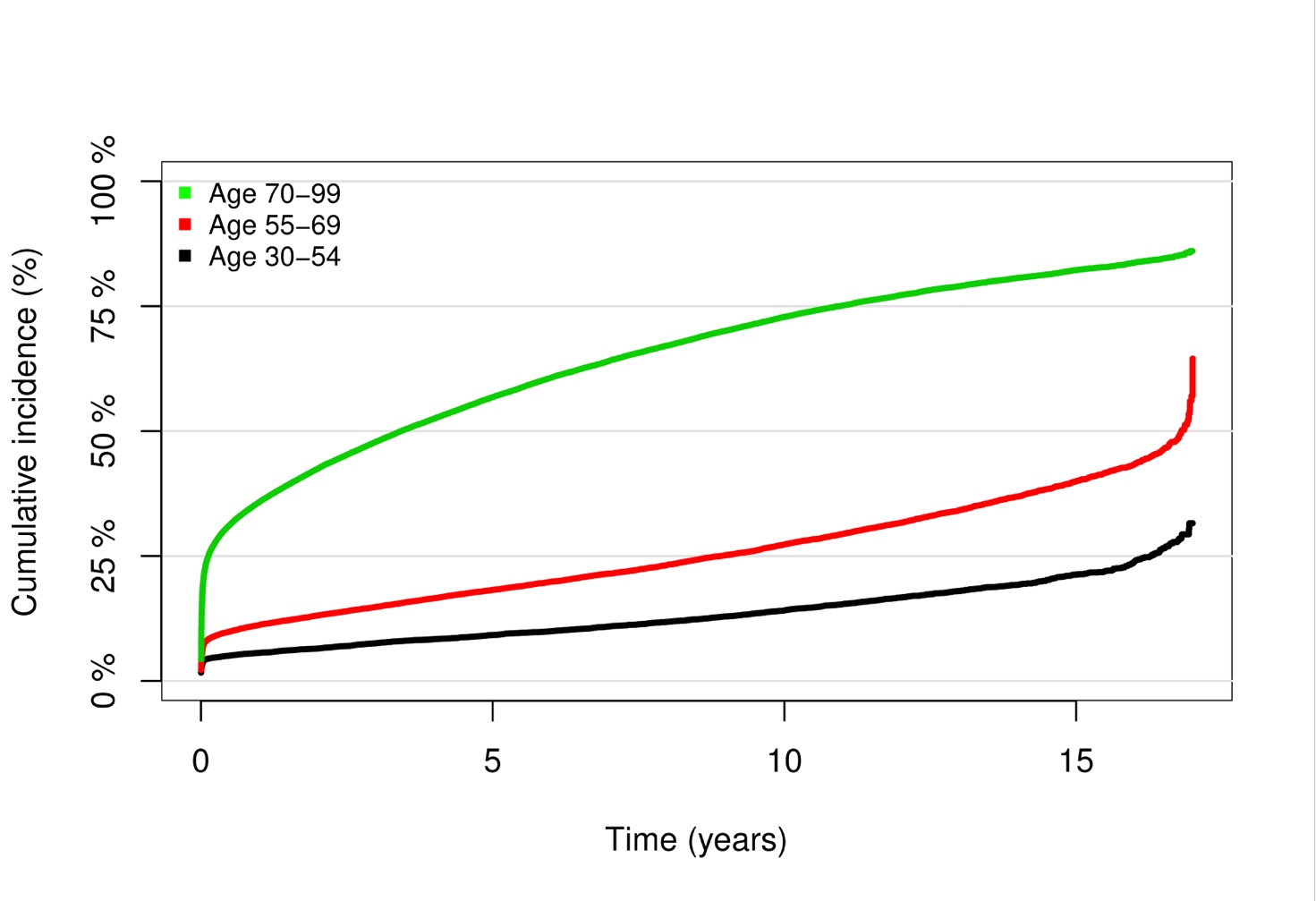
*
