# Supplementary material for: Incidence of new onset cancer in patients with a myocardial infarction – a nationwide cohort study
Source: BMC Cardiovasc Disord. 2018 Oct 22;18:198. doi: 10.1186/s12872-018-0932-z (PMC6196475; doi:10.1186/s12872-018-0932-z)
Supplement: Supplementary file 2 — Table S1. One-year incidence rates of subtypes of cancer per 1,000 person-years according to age group. Table S2A. Incidence rate ratios of cancer associated with an MI adjusted for age, sex and calendar year according to time since MI. Table S2B. Incidence rate ratios of cancer associated with an MI adjusted for age, sex, calendar year, dyslipidemia, hypertension, diabetes, chronic obstructive pulmonary disease and socioeconomic status according to time since MI. Table S3A. Incidence rate ratios of overall cancer and selected subtypes of cancer stratified by age group and adjusted for sex and calendar year. Table S3B. Incidence rate ratios of overall cancer and selected subtypes of cancer stratified by age group and adjusted for sex, calendar year, dyslipidemia, hypertension, diabetes, chronic obstructive pulmonary disease and socioeconomic status. Table S3C. Incidence rate ratios of overall cancer and selected subtypes of cancer 6 months – 17 years post-MI stratified by age group and adjusted for sex, calendar year, dyslipidemia, hypertension, diabetes, chronic obstructive pulmonary disease and socioeconomic status. Table S4. Incidence rate ratios of overall cancer and selected subtypes of cancer stratified by gender. (DOCX 54 kb) [file 12872_2018_932_MOESM2_ESM.docx]

**Supplemental tables**

*Additional file 2: Table S1. One-year incidence rates of subtypes of cancer per 1,000 person-years according to age group.*

|  | Lung | | Colorectal | | LUT | | Breast | | Prostate | |
| --- | --- | --- | --- | --- | --- | --- | --- | --- | --- | --- |
| Age group | Reference  (95% CI)  n | MI  (95% CI)  n | Reference  (95% CI)  n | MI  (95% CI)  n | Reference  (95% CI)  n | MI  (95% CI)  n | Reference  (95% CI)  n | MI  (95% CI)  n | Reference  (95% CI)  n | MI  (95% CI)  n |
| 30-54 | 0.3  (0.3-0.3)  6310 | 0.8  (0.6-1.1)  61 | 0.3  (0.3-0.3) 5100 | 0.5  (0.4-0.7)  39 | 0.0  (0.0-0.1)  924 | 0.1  (0.1-0.3)  10 | 1.7  (1.6-1.7) 16369 | 1.8  (1.3-2.6)  30 | 0.1  (0.1-0.1)  1026 | 0.2  (0.1-0.3)  10 |
| 55-69 | 1.9  (1.9-2.0) 25743 | 3.2  (3.0-3.5)  733 | 1.5  (1.5-1.5) 20040 | 1.7  (1.5-1.9)  388 | 0.4  (0.4-0.4) 5239 | 0.7  (0.6-0.8)  166 | 3.8  (3.8-3.9) 25755 | 3.8  (3.4-4.4)  211 | 2.3  (2.2-2.3) 14720 | 2.4  (2.2-2.7)  420 |
| 70-99 | 3.9  (3.8-3.9) 31149 | 5.7  (5.4-6.0)  1673 | 4.2  (4.1-4.2) 33529 | 4.5  (4.3-4.7) 1319 | 1.2  (1.2-1.3) 9841 | 1.8  (1.7-2.0)  539 | 4.0  (3.9-4.0) 19247 | 3.4  (3.1-3.8)  436 | 6.6  (6.6-6.7) 21055 | 7.0  (6.6-7.4)  1170 |
| Total | 1.6  (1.6-1.6) 63202 | 4.2  (4.0-4.3)  2467 | 1.5  (1.5-1.5) 58669 | 2.9  (2.8-3.1) 1746 | 0.4  (0.4-0.4) 16004 | 1.2  (1.1-1.3)  715 | 2.9  (2.9-2.9) 60371 | 3.4  (3.2-3.7)  677 | 1.9  (1.9-1.9) 36801 | 4.0  (3.8-4.2)  1600 |

** = p<0.05, MI = myocardial infarction, LUT = lower urinary tract, 95% CI = 95% confidence intervals.*

*Additional file 2: Table S2A. Incidence rate ratios of cancer associated with an MI adjusted for age, sex and calendar year according to time since MI.*

|  | **Overall** | **Lung** | **Colorectal** | **LUT** | **Breast** | **Prostate** |
| --- | --- | --- | --- | --- | --- | --- |
| **Time post-MI** | IRR  (CI 95%)  *p* | IRR  (CI 95%)  *p* | IRR  (CI 95%)  *p* | IRR  (CI 95%)  *p* | IRR  (CI 95%)  *p* | IRR  (CI 95%)  *p* |
| **0-1 months** | 4.08  (3.54-4.70)  <0.0001* | 5.25  (3.70-7.45)  <0.0001* | 2.45  (1.91-3.15)  <0.0001* | 4.05  (2.91-6.65)  <0.0001* | 2.12  (0.98-4.58)  0.055 | 3.80  (3.07-4.70)  <0.0001* |
| **1-3 months** | 1.84  (1.57-2.15)  <0.0001* | 2.73  (1.91-3.90)  <0.0001* | 1.96  (1.59-2.41)  <0.0001* | 1.24  (0.80-1.93)  0.336 | 0.86  (0.35-2.11)  0.746 | 1.05  (0.78-1.41)  0.775 |
| **3-6 months** | 1.34  (1.15-1.56)  0.0002* | 1.56  (1.05-2.30)  0.026* | 1.38  (1.12-1.69)  0.002* | 1.65  (1.20-2.27)  0.002* | 0.91  (0.45-1.87)  0.804 | 1.06  (0.83-1.35)  0.646 |
| **6-12 months** | 1.10  (1.00-1.20)  0.052 | 1.23  (0.97-1.57)  0.090 | 0.98  (0.86-1.12)  0.773 | 1.35  (1.11-1.64)  0.003* | 0.83  (0.55-1.26)  0.388 | 0.91  (0.79-1.05)  0.207 |
| **1-5 years** | 1.04  (0.99-1.10)  0.162 | 1.28  (1.12-1.47)  0.0003* | 0.90  (0.83-0.97)  0.009* | 1.12  (1.00-1.27)  0.059 | 0.79  (0.61-1.01)  0.055 | 0.81  (0.75-0.88)  <0.0001* |
| **5-10 years** | 1.09  (1.02-1.17)  0.008* | 1.35  (1.15-1.60)  0.0004* | 0.97  (0.88-1.06)  0.481 | 1.27  (1.10-1.47)  0.001* | 0.87  (0.63-1.18)  0.361 | 0.93  (0.85-1.02)  0.137 |
| **10-17 years** | 1.09  (0.98-1.20)  0.110 | 1.37  (1.05-1.79)  0.022* | 0.90  (0.77-1.05)  0.190 | 1.36  (1.09-1.69)  0.006* | 1.14  (0.72-1.79)  0.583 | 0.85  (0.74-0.97)  0.018* |

**=p<0.05, IRR = incidence rate ratio, LUT = lower urinary tract, 95% CI = 95% confidence intervals.*

*Additional file 2: Table S2B. Incidence rate ratios of cancer associated with an MI adjusted for age, sex, calendar year, dyslipidemia, hypertension, diabetes, chronic obstructive pulmonary disease and socioeconomic status according to time since MI.*

|  | **Overall** | **Lung** | **Colorectal** | **LUT** | **Breast** | **Prostate** |
| --- | --- | --- | --- | --- | --- | --- |
| **Time post-MI** | IRR  (CI 95%)  *p* | IRR  (CI 95%)  *p* | IRR  (CI 95%)  *p* | IRR  (CI 95%)  *p* | IRR  (CI 95%)  *p* | IRR  (CI 95%)  *p* |
| **0-1 months** | 3.81  (3.21-4.52)  <0.0001* | 4.22  (2.81-6.35)  <0.0001* | 2.35  (1.34-4.13)  0.003* | 3.83  (2.25-6.51)  <0.0001* | 2.09  (1.00-4.37)  0.052 | 3.74  (2.55-5.51)  <0.0001* |
| **1-3 months** | 1.72  (1.42-2.08)  <0.0001* | 2.17  (1.43-3.30)  0.0003* | 1.89  (1.18-3.01)  0.008* | 1.17  (0.58-2.39)  0.659 | 0.85  (0.36-2.01)  0.711 | 1.03  (0.60-1.77)  0.924 |
| **3-6 months** | 1.25  (1.04-1.50)  0.016* | 1.24  (0.79-1.96)  0.351 | 1.33  (0.84-2.10)  0.229 | 1.55  (0.93-2.59)  0.090 | 0.90  (0.45-1.79)  0.759 | 1.04  (0.67-1.61)  0.874 |
| **6-12 months** | 1.03  (0.92-1.15)  0.653 | 0.99  (0.75-1.32)  0.944 | 0.95  (0.70-1.28)  0.719 | 1.27  (0.93-1.75)  0.135 | 0.82  (0.55-1.22)  0.327 | 0.89  (0.68-1.16)  0.390 |
| **1-5 years** | 0.98  (0.92-1.04)  0.496 | 1.04  (0.89-1.22)  0.608 | 0.87  (0.73-1.04)  0.129 | 1.06  (0.87-1.29)  0.553 | 0.77  (0.61-0.98)  0.034* | 0.80  (0.69-0.93)  0.004* |
| **5-10 years** | 1.03  (0.95-1.11)  0.485 | 1.12  (0.92-1.37)  0.259 | 0.93  (0.75-1.16)  0.533 | 1.20  (0.95-1.51)  0.130 | 0.85  (0.63-1.15)  0.285 | 0.92  (0.78-1.09)  0.339 |
| **10-17 years** | 1.02  (0.91-1.16)  0.717 | 1.14  (0.83-1.56)  0.416 | 0.87  (0.61-1.23)  0.425 | 1.28  (0.90-1.81)  0.176 | 1.11  (0.72-1.73)  0.632 | 0.84  (0.66-1.09)  0.187 |

**=p<0.05, IRR = incidence rate ratio, LUT = lower urinary tract, 95% CI = 95% confidence intervals.*

*Additional file 2: Table S3A. Incidence rate ratios of overall cancer and selected subtypes of cancer stratified by age group and adjusted for sex and calendar year.*

|  | Overall | Lung | Colorectal | LUT | Breast | Prostate |
| --- | --- | --- | --- | --- | --- | --- |
| Age group | IRR  (CI 95%) *p* | IRR  (CI 95%) *p* | IRR  (CI 95%) *p* | IRR  (CI 95%) *p* | IRR  (CI 95%) *p* | IRR  (CI 95%) *p* |
| 30-54 | 1.55  (1.34-1.78)  <0.0001* | 2.43  (1.78-3.32)  <0.0001* | 1.70  (1.24-2.32)  0.001* | 2.20 (1.07-4.43)  0.032* | 0.96  (0.74-1.22)  0.727 | 1.13 (0.62-2.06)  0.695 |
| 55-69 | 1.28  (1.18-1.38)  <0.0001* | 1.66  (1.44-1.92)  <0.0001* | 1.06 (0.97-1.17)  0.180 | 1.58 (1.33-1.88)  <0.0001* | 0.97  (0.82-1.15)  0.732 | 0.94 (0.84-1.05)  0.280 |
| 70-99 | 1.13  (1.11-1.16)  <0.0001* | 1.32  (1.16-1.50)  <0.0001* | 1,04  (0.99-1.10)  0.140 | 1.27 (1.15-1.41)  <0.0001* | 0.87  (0.80-0.95)  0.001* | 1.02 (0.96-1.08)  0.457 |

**=p<0.05, IRR = incidence rate ratio, LUT = lower urinary tract, 95% CI = 95% confidence interval.*

*Additional file 2: Table S3B. Incidence rate ratios of overall cancer and selected subtypes of cancer stratified by age group and adjusted for sex, calendar year, dyslipidemia, hypertension, diabetes, chronic obstructive pulmonary disease and socioeconomic status.*

|  | Overall | Lung | Colorectal | LUT | Breast | Prostate |
| --- | --- | --- | --- | --- | --- | --- |
| Age group | IRR  (CI 95%) *p* | IRR  (CI 95%) *p* | IRR  (CI 95%) *p* | IRR  (CI 95%) *p* | IRR  (CI 95%) *p* | IRR  (CI 95%) *p* |
| 30-54 | 1.15  (0.88-1.50)  0.305 | 1.29  (0.87-1.93)  0.210 | 1.34  (0.96-1.87)  0.087 | 1.68 (0.72-3.92)  0.227 | 0.87  (0.54-1.40)  0.573 | 0.80 (0.47-1.36)  0.408 |
| 55-69 | 1.09  (1.04-1.15)  0.0004* | 1.21  (1.05-1.39)  0.008* | 0.95 (0.84-1.07)  0.364 | 1.29 (1.11-1.50)  0.001* | 0.90  (0.78-1.05)  0.179 | 0.80 (0.71-0.89)  <0.0001* |
| 70-99 | 1.09  (1.04-1.14)  <0.0001* | 1.13  (1.05-1.21)  0.0005* | 1.01  (0.95-1.06)  0.849 | 1.25 (1.13-1.37)  <0.0001* | 0.87  (0.78-0.97)  0.011* | 1.02 (0.95-1.09)  0.630 |

**=p<0.05, IRR = incidence rate ratio, LUT = lower urinary tract, 95% CI = 95% confidence interval.*

*Additional file 2: Table S3C. Incidence rate ratios of overall cancer and selected subtypes of cancer 6 months – 17 years post-MI stratified by age group and adjusted for sex, calendar year, dyslipidemia, hypertension, diabetes, chronic obstructive pulmonary disease and socioeconomic status.*

|  | Overall | Lung | Colorectal | LUT | Breast | Prostate |
| --- | --- | --- | --- | --- | --- | --- |
| Age group | IRR  (CI 95%) *p* | IRR  (CI 95%) *p* | IRR  (CI 95%) *p* | IRR  (CI 95%) *p* | IRR  (CI 95%) *p* | IRR  (CI 95%) *p* |
| 30-54 | 1.09  (0.81-1.46)  0.589 | 1.22  (0.78-1.91)  0.387 | 1.32  (0.92-1.89)  0.130 | 1.87 (0.79-4.43)  0.156 | 0.87  (0.53-1.43)  0.581 | 0.69 (0.37-1.27)  0.229 |
| 55-69 | 1.02  (0.96-1.07)  0.588 | 1.12  (1.01-1.24)  0.036* | 0.89 (0.75-1.04)  0.138 | 1.15 (0.97-1.36)  0.114 | 0.89  (0.76-1.04)  0.136 | 0.80 (0.71-0.89)  <0.0001* |
| 70-99 | 1.01  (0.97-1.06)  0.581 | 1.04  (0.97-1.12)  0.232 | 0.93  (0.88-0.99)  0.021* | 1.20 (1.09-1.33)  0.0003* | 0.84  (0.72-0.98)  0.025* | 0.94 (0.88-1.01)  0.105 |

**=p<0.05, IRR = incidence rate ratio, LUT = lower urinary tract, 95% CI = 95% confidence interval.*

*Additional file 2: Table S4. Incidence rate ratios of overall cancer and selected subtypes of cancer stratified by gender.*

|  | Overall | Lung | Colorectal | LUT | Breast | Prostate |
| --- | --- | --- | --- | --- | --- | --- |
| Sex | IRR  (CI 95%) *p* | IRR  (CI 95%) *p* | IRR  (CI 95%) *p* | IRR  (CI 95%) *p* | IRR  (CI 95%) *p* | IRR  (CI 95%) *p* |
| Women |  |  |  |  |  |  |
| A | 1.17 (1.12-1.22) <0.0001* | 1.77 (1.62-1.94) <0.0001* | 1.04 (0.96-1.13) 0.343 | 1.54 (1.29-1.83) <0.0001* | 0.87 (0.80-0.95) 0.002* | - |
| AB | 1.08 (1.00-1.15) 0.040* | 1.25 (1.08-1.44) 0.003* | 1.00 (0.82-1.21) 0.977 | 1.41 (1.11-1.80) 0.005* | 0.86 (0.73-1.00) 0.052 | - |
| ABC | 0.99 (0.92-1.06) 0.718 | 1.12 (0.96-1.30) 0.153 | 0.91 (0.75-1.11) 0.358 | 1.34 (1.03-1.75) 0.032* | 0.83 (0.70-0.99) 0.041* | - |
| Men |  |  |  |  |  | - |
| A | 1.09 (1,06-1.12) <0.0001* | 1.27 (1.20-1.35) <0.0001* | 0.97 (0.91-1.03) 0.298 | 1.24 (1.14-1.34) <0.0001* | - | 0.91 (0.86-0.97) 0.003* |
| AB | 1.04 (0.97-1.10) 0.275 | 1.12 (0.94-1.33) 0.195 | 0.94 (0.83-1.06) 0.300 | 1.17 (1.01-1.35) 0.041* | - | 0.90 (0.80-1.02) 0.108 |
| ABC | 0.97 (0.91-1.03) 0.262 | 1.04 (0.87-1.24) 0.648 | 0.88 (0.77-1.00) 0.057 | 1.11 (0.95-1.30) 0.205 | - | 0.85 (0.77-0.85) 0.005* |

**=p<0.05, IRR = incidence rate ratio, LUT = lower urinary tract, 95% CI = 95% confidence interval.
A adjusted for age and calendar year.
B adjusted for dyslipidemia, hypertension, diabetes, chronic obstructive pulmonary disease and socioeconomic status.
C adjusted the first 6 months after an MI.*
